# Treatment Outcomes for Malignant Peripheral Lung Tumours: A Prospective Single-Centre Comparison of Thermal Ablation, Surgery, and Radiotherapy

**DOI:** 10.3390/medicina61111972

**Published:** 2025-11-03

**Authors:** Aurimas Mačionis, Gertrūda Maziliauskienė, Rūta Dubeikaitė, Ieva Balčiūnaitė, Grytė Galnaitienė, Lina Padervinskienė, Jurgita Matulionė, Skaidrius Miliauskas, Erika Korobeinikova, Laimonas Jaruševičius, Irena Nedzelskienė, Edita Mišeikytė-Kaubrienė, Donatas Vajauskas, Marius Žemaitis

**Affiliations:** 1 Department of Radiology, Medical Academy, Lithuanian University of Health Sciences, LT-44307 Kaunas, Lithuania; gertruda.maziliauskiene@kaunoklinikos.lt (G.M.); ruta.dubeikaite@kaunoklinikos.lt (R.D.); ieva.balciunaite@kaunoklinikos.lt (I.B.); gryte.galnaitiene@kaunoklinikos.lt (G.G.); lina.padervinskiene@kaunoklinikos.lt (L.P.); donatas.vajauskas@kaunoklinikos.lt (D.V.); 2 Department of Pulmonology, Medical Academy, Lithuanian University of Health Sciences, LT-44307 Kaunas, Lithuania; jurgita.matulione@kaunoklinikos.lt (J.M.); marius.zemaitis@kaunoklinikos.lt (M.Ž.); 3 Department of Oncology and Hematology, Medical Academy, Lithuanian University of Health Sciences, LT-44307 Kaunas, Lithuania; erika.korobeinikova@kaunoklinikos.lt (E.K.); laimonas.jarusevicius@kaunoklinikos.lt (L.J.); 4 Department of Dental and Oral Diseases, Hospital of Lithuanian University of Health Sciences Kauno Klinikos, LT-50161 Kaunas, Lithuania; irena.nedzelskiene@lsmu.lt; 5 Department of Radiology, Faculty of Medicine, Vilnius University, LT-03101 Vilnius, Lithuania; edita.miseikyte@mf.vu.lt

**Keywords:** non-small cell lung cancer, lung metastases, surgery, image-guided thermal ablation, microwave ablation, cryoablation, stereotactic body radiotherapy, treatment outcomes, cancer recurrence, quality-of-life

## Abstract

*Background and Objectives*: Lung cancer and pulmonary metastases are major causes of cancer-related mortality. Surgery is a standard curative approach, but many patients are ineligible due to age, comorbidities, or treatment preference. This study aimed to evaluate the safety, effectiveness, and quality-of-life outcomes of thermal ablation versus surgery and stereotactic body radiotherapy (SBRT) for malignant lung lesions. *Materials and Methods*: A prospective, non-randomized study was conducted on 68 patients with primary or metastatic lung tumours treated by surgery (n = 19), SBRT (n = 29), or thermal ablation (n = 20). The key outcomes included recurrence rates and patterns, disease-free and overall survival, complications, hospitalization, and health-related quality of life (HRQoL). *Results*: Surgery demonstrated the lowest total and regional recurrence rates (21.1% and 10.5%, respectively), significantly lower than SBRT (57.1% and 42.9%, respectively; *p* < 0.05). Additionally, surgery led to the longest disease-free survival but was associated with the highest complication rate (78.9%) and the greatest HRQoL decline. SBRT had fewer complications (17.2%) and moderate HRQoL outcomes. Thermal ablation showed no significant differences in recurrence (45.0% of total recurrence) or survival compared to surgery or SBRT, with a moderate complication rate (45.0%) and the most favorable HRQoL outcomes. Major complications were rare and comparable across all groups. *Conclusions*: Thermal ablation demonstrated comparable disease control and quality-of-life outcomes to SBRT, with lower complication rates. While surgery remains superior in local disease control, its invasiveness and impact on quality of life underscore the importance of minimally invasive treatments in multidisciplinary management of malignant lung lesions.

## 1. Introduction

In 2022, lung cancer led global cancer morbidity and mortality with nearly 2.5 million new cases and over 1.8 million deaths, accounting for roughly 12.4% of all cancer diagnoses and 18.7% of cancer-related deaths worldwide [1]. Lung cancer has a range of risk factors, combining genetic, environmental, and occupational influences, such as smoking, second-hand smoking, both indoor and outdoor air pollution, ionizing radiation, and exposure to asbestos, arsenic, chromium, silica, and vinyl chloride [2]. A meaningful proportion of lung cancer cases and deaths could be prevented with adequate awareness of its risk factors, and stricter government policies and interventions to target environmental and occupational components. Although an increasing proportion of lung cancer cases are now diagnosed at stage I—reflecting the impact of increased screening—a large number of patients are still diagnosed at advanced stages (28% at stage III and 38% at stage IV), when curative options are limited [3]. Notably, about half of NSCLC (non-small cell lung cancer) patients are aged 70 or older [4], and many present with comorbidities such as chronic obstructive pulmonary disease and heart failure or have poor performance status, rendering approximately a fifth of patients inoperable [4,5,6].

The lungs are among the most common sites for metastases, with up to 54% of cancers originating in other parts of the body spreading to the lungs [7,8]. In adults, colorectal cancer is the most common origin of pulmonary metastases, followed by breast, renal, head and neck cancers, and uterine leiomyosarcoma [8,9]. The lungs are a common site for both synchronous (diagnosed at or around the time of initial cancer diagnosis) and metachronous (developed during follow-ups) metastatic disease, and the incidence of cancers presenting with synchronous spread to the lungs is increasing over time [10]. The presence of lung metastases is a crucial factor influencing treatment choices and disease prognosis. A multimodal approach forms the foundation of lung metastasis treatment, with systemic chemotherapy remaining the cornerstone. Recent advances in treatment strategies—including molecular-targeted therapies and immunotherapy—have significantly enhanced its effectiveness [11]. Additionally, evidence suggests that surgical resection may provide a substantial survival benefit in carefully selected patients [9].

Surgical resection remains the key component of curative treatment for early-stage non-small cell lung cancer and resectable pulmonary metastases. Surgery offers high local control and long-term survival, particularly in operable patients with good performance status [12]. However, a substantial proportion of patients are unfit or unwilling to undergo the surgery. As a non-invasive alternative, stereotactic body radiotherapy (SBRT) delivers ablative radiation doses with reliable local control, especially in peripheral tumours, but carries a risk of significant toxicity, particularly for centrally located lesions [12,13]. Image-guided thermal ablation (IGTA), including radiofrequency ablation (RFA), microwave ablation (MWA), and cryoablation (CA) has emerged as a minimally invasive alternative. It combines local tumour destruction with preservation of healthy lung tissue and is particularly valuable for high-risk or inoperable patients [12,14,15]. While growing evidence supports the efficacy of thermal ablation, its comparative effectiveness against standard radical treatments remains to be clearly defined. The aim of this non-randomized study is to evaluate the effectiveness and safety of minimally invasive ablative therapy for malignant pulmonary lesions and to compare it with standard radical treatment methods—surgical resection and SBRT—reflecting real-world clinical management experience.

## 2. Materials and Methods

### 2.1. Study Overview

A prospective, non-randomised comparative study was conducted at Kauno Klinikos Hospital, a tertiary care centre affiliated with the Lithuanian University of Health Sciences. The study received approval from the Kaunas Regional Biomedical Research Ethics Committee in November 2022 (protocol number: 2022-BE-10-0015).

Eligible participants were adults with a confirmed diagnosis of systemic-therapy-naïve biopsy-proven NSCLC (stages Tis-T2) or oligometastatic peripheral lung disease with five or fewer lesions. All included lesions were ≤3 cm is size, accessible for surgery, ablation, or SBRT, and without local nodal or distant metastatic disease on initial imaging. Patients with central lung tumors (<2 cm from the main bronchi or hilum), more than five or diffuse lung lesions, or prior local treatment to the same lesion were not included in the study. Additional exclusion criteria included major comorbidities, physical or social limitations precluding trial participation, contraindications to general anaesthesia, the need for continuous antiplatelet therapy, or evidence of severe coagulopathy. All candidates were assessed in multidisciplinary team (MDT) meetings consisting of a pulmonologist, interventional radiologist, radiation therapist, thoracic surgeon, and oncologist. The MDT comprehensively evaluated performance status, comorbidities (including pulmonary function), disease characteristics to determine the feasibility of surgery, SBRT, or ablation. Only patients deemed suitable by the MDT were offered participation, with all appropriate treatment options presented. The final treatment decision was made jointly, incorporating MDT recommendations and the patient’s individual preference. Written informed consent to undergo the proposed therapy and to participate in the study was obtained from all participants. This individualized decision-making process ensured that treatment allocation was guided not only by clinical feasibility but also by patient-specific needs and preferences.

**Figure 1:**
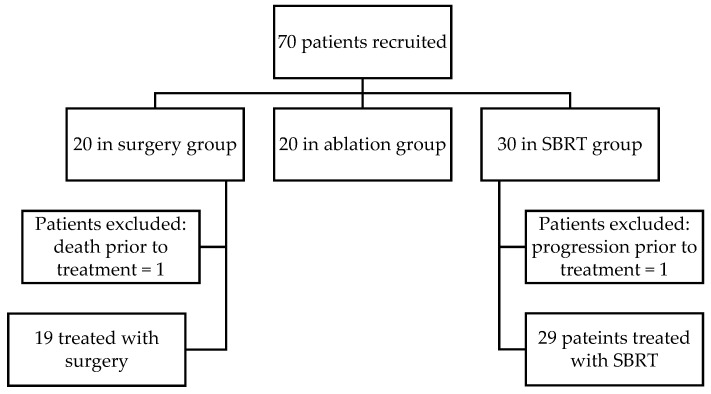
Patient enrollment flowchart.

Patient recruitment was carried out continuously over a 2-year period (from November 2022 to November 2024). Of the 70 patients who met the inclusion criteria, 68 were enrolled in the study. One patient in the surgery group died prior to surgery, and one patient in the SBRT group developed systemic disease progression before initiation of SBRT and was no longer subjected to radical treatment. The patient enrollment flowchart is presented in Figure 1. The sample size was determined by the number of eligible patients meeting the inclusion criteria during recruitment timeframe, reflecting a pragmatic, real-world cohort design.

Clinical data related to the patient, tumour, and procedure were gathered from patients and their clinical and imaging records. Patient-related characteristics included age, sex, ECOG status, health-related quality of life (HRQoL), and overall survival (OS). Tumour-related characteristics encompassed tumour histology and the number of treated lesions. Procedure-specific information included the intended type of therapy, therapy parameters (irradiation dose and fractions, surgical approach and extent, ablation duration and output power), completion date, occurrence of complications (including type, severity grade, and management approach), and length of hospitalisation. Follow-up imaging provided details on the timing, type, and location of disease recurrence, as well as disease-free survival (DFS). Due to the nature of the interventions, neither participants nor investigators were blinded to treatment allocation.

### 2.2. Treatment

#### 2.2.1. Surgery

The surgical group included 19 patients. All treated lesions were classified as T1 on baseline imaging. All surgeries were performed by experienced thoracic surgeons under general endotracheal anaesthesia with the aim of complete cancer removal and ensuring negative oncologic margins. Eighteen patients had primary lung tumours and were treated with anatomical resections, including 17 cases of lobectomies (RUL 9, RLL 3, LUL 1, LLL 4) and 1 case of segmentectomy (LUL segments 1–3). All anatomical resections were carried out with accompanying lymphonodectomy. One patient was diagnosed with a metastatic lesion and treated with atypical resection, which did not include lymph node dissection. All surgeries were carried out via an open thoracotomy approach; in 6 cases, mini thoracotomies were performed.

#### 2.2.2. SBRT

The SBRT group included 29 patients with 33 treated lesions. Three lesions were classified as T2 on initial CT imaging due to visceral pleural involvement, although all measured <3 cm; all other primary lesions were classified as T1. For treatment simulation 2.5 mm slice thickness, non-contrast 4D-CT scan of the chest was acquired using the Varian Real-Time Position Management System (Varian Medical Systems, Palo Alto, CA, USA) to capture the complete respiratory cycle. The gross tumour volume (GTV) was delineated in all 4D-CT phases using lung parenchyma windows; the clinical target volume (CTV) matched the GTV, and the internal target volume (ITV) was generated by encompassing the CTV across all respiratory phases. A 5 mm isotropic expansion of ITV created the planning target volume (PTV). Treatment planning followed RTOG 0813 [16] and 0915 [17] recommendations for organ-at-risk (OAR) constraints. Four-dimensional cone-beam CT (CBCT) was used prior to each fraction for setup verification and correction. SBRT was delivered with multileaf collimator-shaped conformal arcs, ensuring prescription coverage of the 95% isodose line, with doses >105% confined within the PTV and a conformity index target of <1.2.

Treatments were delivered with a TrueBeam™ linear accelerator (Varian Medical Systems, Palo Alto, CA, USA). SBRT was administered using fractionation schemes tailored by the radiation oncologist based on tumour size and proximity to critical structures. The biological effective dose (BED_10_) was calculated by the formula, BEDα/β = nd (1 + d/α/β), wherein n is the fraction number, d is the dose per fraction, with α/β = 10 Gy. The median BED_10_ was 100 Gy (range, 72–151.2 Gy). Table 1 summarizes the dose and fractionation regimens used, while Table 2 presents the baseline dosimetric characteristics of the study population. The most common regimens were 50–55 Gy in 3–5 fractions (n = 23, 69.7%) and 60 Gy in 8 fractions (n = 4, 12.1%).

#### 2.2.3. Ablation

The ablation group consisted of 20 patients with 25 treated lesions. Eighteen patients underwent microwave ablation and 2 received cryoablation. All ablation procedures were performed by a skilled interventional radiologist under CT guidance using a Revolution Ascend 64-slice CT scanner (GE Healthcare, Louisville, KY, USA). A low-dose CT scan protocol, which includes a tube voltage of 100.0 kV, tube current of 50.0–100.0 mA, and a 1.25 mm slice thickness, was utilised for planning the intervention and monitoring intra-procedural ablation zone. Patient positioning during the procedure was adjusted according to lesion location to prone, supine, or lateral decubitus. All ablation sessions were carried out under general anaesthesia.

Microwave ablation was accomplished using the TATO2 system (Biomedical Srl, Florence, Italy), approved under the 93/42/EEC directive for medical devices. The system operated at a frequency of 2.4–2.483 GHz with an ablative output power of 30 W. The mean ablation time was 21 ± 9 min (range: 10–40 min). Procedures were conducted using 15 G coaxial introducer needles and 17 G antennas. In most cases, a single antenna was applied using either an overlapping (18/23) or single-position (3/23) technique, while two procedures required the use of dual antennas to ensure adequate ablation coverage.

Cryoablation was performed using the CryoCare Touch™ ablation system (Varian Medical Systems, Austin, TX, USA), approved under the 93/42/EEC directive. Cryoprobes with a 1.7 mm shaft diameter and 15–20 cm shaft length were employed, with one or multiple probes used per session depending on the lesion size. The procedure consisted of three consecutive freeze–thaw cycles, utilising passive thawing between the freezing phases. In both cases, the active freezing phase lasted a total of 23 min: one 3 min cycle followed by two 10 min cycles. Ice ball formation was monitored throughout the procedure to ensure adequate lesion coverage and sufficient safety margins.

The target ablation margin was at least 8–10 mm for metastatic lesions and 15 mm for primary tumors in both MWA and cryoablation procedures. All ablation sessions were completed with tract sealing to reduce the risk of complications. The patient’s clotted venous blood was gradually injected into all intervention sites while withdrawing the coaxial needles in small increments, allowing time for the clot to set.

### 2.3. Follow-Up

The standard follow-up protocol included chest CT scans at 1 and 3 months, followed by chest–abdomen CT (with pelvic imaging for patients with primary abdominal or pelvic malignancies) at 6, 12, 18, and 24 months post-treatment. The imaging protocol comprised unenhanced and contrast-enhanced (arterial and venous phases) CT scans with a 1.25 mm slice thickness.

In cases where local recurrence was uncertain on CT, FDG-PET/CT (preferably performed at least 6 months after treatment completion) or a targeted biopsy was conducted for further evaluation. Follow-up imaging of ablated lesions was assessed in accordance with ECIO-ESOI evidence and consensus-based recommendations [18]. Indicators of local recurrence on CT included an increase in ablation zone size, new contrast media uptake (≥15 HU), and the presence of a nodular, irregular, asymmetrically solid area within or along the edge of the ablation zone. On PET/CT, local relapses were suggested by new FDG uptake in a solid region at the ablation margin or within the irradiated area.

One patient treated with microwave ablation showed an unchanged contrast enhancement pattern and lesion size on the 1-month follow-up CT, indicating unsuccessful ablation. The lesion was subsequently re-treated with MWA shortly after, achieving full devascularization. The date of the re-treatment was considered the completion of therapy and served as the baseline for patient follow-up.

### 2.4. Outcomes

The chosen outcomes were designed to capture both oncologic effectiveness and patient-centered measures, emphasizing individualized treatment responses to guide a personalized management approach.

#### 2.4.1. Complications

Complications in all participants across the three treatment groups were classified using the same Cardiovascular and Interventional Radiological Society of Europe (CIRSE) criteria [19] to enable direct comparison:

- Grade 1: Complication during the procedure that could be solved within the same session; no additional therapy, no post-procedure sequelae, no deviation from the normal post-therapeutic course.
- Grade 2: Prolonged observation including overnight stay (as a deviation from the normal post-therapeutic course < 48 h); no additional post-procedure therapy, no post-procedure sequelae.
- Grade 3: Additional post-procedure therapy or prolonged hospital stay (>48 h) required; no post-procedure sequelae.
- Grade 4: Complication causing a permanent mild sequelae (resuming work and independent living).
- Grade 5: Complication causing a permanent severe sequelae (requiring ongoing assistance in daily life).
- Grade 6: Death.

The typical complication monitoring for ablation patients included a chest CT scan 10 min and 24 h after (before discharge) the ablation. Daily or every-other-day chest X-rays were performed during the initial postoperative management of surgical patients. SBRT patients had no specific post-treatment imaging observation protocol. If indicated, additional chest X-rays or CTs were performed for any patient suspected of complications.

Significant pneumothorax was defined as large at presentation, progressive, or symptomatic, and those cases were drained. All surgery patients were drained post-operatively as per the surgery protocol.

#### 2.4.2. Recurrence

Recurrence was assessed through follow-up imaging. Local control was defined as the absence of tumour progression at the primary site, including the ablation, or post-radiation zone margins. Regional recurrence was characterised by cancer reappearing in other areas of the lungs, within the pleura or in thoracic lymph node stations. Any newly detected lesion beyond these regions was classified as a distant recurrence.

#### 2.4.3. Survival

The analysed indicators were overall survival and disease-free survival. Overall survival was defined as the duration from the completion of the intended therapy to death from any cause. Disease-free survival was measured as the time from the completion of the intended therapy to either disease recurrence in any form or death from any cause, whichever occurred first.

#### 2.4.4. Health-Related Quality of Life Assessment

All enrolled patients were subjected to report disease-related quality of life using the SF-36 questionnaire [20] (Lithuanian translation). The questionnaire consisted of 36 questions categorised into eight domains: physical functioning, role limitations due to physical health, social functioning, mental health, role limitations due to emotional health, vitality, bodily pain, and overall health. Each domain was scored from 0 to 100, with 0 representing the poorest health status and 100 indicating the best possible health status. Patients completed the SF-36 questionnaire twice: before treatment and one month after completing the intended therapy. Treatment-induced changes in physical and psychological health were determined by comparing pre- and post-treatment scores for each domain. Positive values indicated improvement, while negative values reflected a decline in the reported domain. Changes across all eight domains were analyzed within each treatment group and compared across all three groups to evaluate the overall impact of therapy on quality of life. This integration of patient-reported outcomes into the evaluation process reflects a personalized approach, ensuring that treatment effectiveness was assessed not only by clinical outcomes but also by individual patient experience.

#### 2.4.5. Statistical Analysis

Statistical analysis of the data was performed using software packages for the storage and analysis of data, SPSS 30.0 (IBM, Armonk, NY, USA). The Kolmogorov–Smirnov test was used for the determination of quantitative data distribution. When the distribution of variables was normal, Student’s t-test was used to compare the quantitative sizes of two independent samples. The Mann–Whitney U test was used to compare non-normally distributed variables. One-way analysis of variance (ANOVA) was used to compare more than two independent groups. The least significant difference (Bonferroni) post hoc test was used for multiple paired comparisons. The Kruskal–Wallis test was used to compare non-normally distributed variables. The interdependence of qualitative evidence was evaluated by chi-square χ^2^ criteria. Depending on the sample size, exact (for small size) and asymptomatic criteria were used. The overall survival and disease-free survival were shown in Kaplan–Meier curves, which are commonly used to analyse time-to-event data, such as the time until death or the time until a specific event occurs. Survival analysis was performed using the Gehan–Breslow–Wilcoxon method, which assigns greater weight to events occurring at earlier time points, an approach particularly appropriate for our study context. Logistic regression analysis was performed to determine the odds ratio predictive value. Differences between groups were considered significant when the level of significance *p* was <0.05. Data in the text is presented as count (%) or median (IQR) values. Primary analyses were pre-specified to compare the three treatment modalities (surgery, SBRT, and ablation) across main outcome measures (recurrence, DFS, complications, hospitalization, and HRQoL), while subgroup comparisons were exploratory.

## 3. Results

The clinical characteristic of the treated population is presented in Table 3. The study included 68 patients, 57.4% of whom were men and 42.6% of whom were women. The surgery group comprised 19 patients (27.9%), SBRT—29 patients (42.7%), and ablation—20 patients (29.4%). The mean age of the study participants was 70.2 ± 8.9 years and did not differ between the treatment groups (*p* = 0.172). The majority of patients had an ECOG Performance Status score of 0 (32.4%) or 1 (61.8%), indicating no or mild activity restrictions. The SBRT group had the highest proportion of ECOG 1 and all ECOG 2 patients, but no significant differences were noticed between the groups (*p* = 0.092).

In total, 77 malignant lesions were treated; their histological characteristics are included in Table 3. Primary NSCLC was most commonly represented by adenocarcinoma (39 lesions), followed by squamous cell carcinoma (16 lesions), while colorectal cancer (CRC) was the most frequent source of metastatic lesions (8 lesions). Primary NSCLC was significantly more likely to be treated with surgery (94.7%) or SBRT (75.8%), whereas ablation was most commonly used for metastatic disease, with 52.0% of lesions being secondary in origin (*p* = 0.002).

### 3.1. Recurrence

The median time for CT follow-up was similar between the groups (Table 4).

Disease recurrence was detected in 29 patients (43.3%), with 14 cases involving multiple recurrence sites identified through follow-up imaging. Table 5 provides a detailed overview of the incidence and pattern of recurrence within the study population. One patient passed away shortly following the completion of SBRT without having any follow-up CT imaging and, thus, is excluded from the recurrence analysis.

Total recurrence was lowest in the surgery group (21.1%) and highest in the SBRT group (57.1%), with a significant difference between these groups (*p* = 0.049). Total recurrence in the ablation group (45.0%) did not differ significantly from either SBRT or surgery. Local recurrence was assessed only in the SBRT and ablation groups, with no statistically significant difference observed between them. Of the recurrent lesions, 11 (19.3%) were primary tumours and 5 (8.8%) were metastases, showing no difference in recurrence pattern (*p* = 0.585). In the ablation group, 5 of 6 local recurrences were successfully treated with repeat ablation, whereas reirradiation following SBRT was successfully performed in only one case.

Regional recurrence occurred in 26.9% of patients, most commonly in the SBRT group (42.9%) and significantly more often than in the surgery group (10.5%; *p* = 0.039). The ablation group showed an intermediate rate of regional recurrence (20.0%), without significant difference from either SBRT or surgery groups. Patients treated with SBRT had a fourfold increased risk of regional recurrence compared to those treated with surgery or ablation (OR 4.125; 95% CI: 1.309–12.996; *p* = 0.012). The mean time to regional recurrence did not differ between the SBRT and ablation groups: 17.1 ± 7.0 months for surgery, 13.3 ± 8.2 months for SBRT, and 16.0 ± 6.1 months for ablation (χ^2^ = 4.39, *p* = 0.111).

Distant recurrence occurred in 20.9% of the study population, with no significant differences between treatment groups. Disease spread was most commonly observed in the liver (n = 5) and bones (n = 4), followed by lymph nodes (n = 3), peritoneum (n = 2), brain (n = 2), adrenals, and thyroid (n = 1 each).

### 3.2. Overall Survival

**Figure 2:**
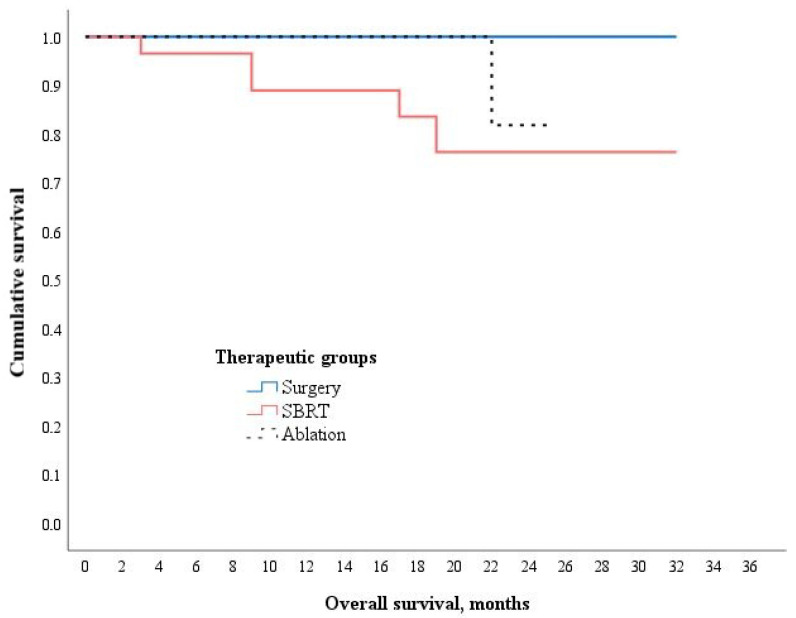
Kaplan–Meier curve for overall survival among treatment groups. χ^2^ (2, N = 68) = 6.237, *p* = 0.044; Wilcoxon (Gehan) Statistic.

No treatment-related deaths occurred among the study participants. Overall, 7 patients (10.3%) died during the follow-up period—5 in the SBRT group and 2 in the ablation group. Pairwise comparisons showed no statistically significant differences in overall survival between the treatment groups: surgery vs. SBRT (*p* = 0.072), surgery vs. ablation (*p* = 0.343), and SBRT vs. ablation (*p* = 0.071) (Figure 2).

### 3.3. Disease-Free Survival

**Figure 3:**
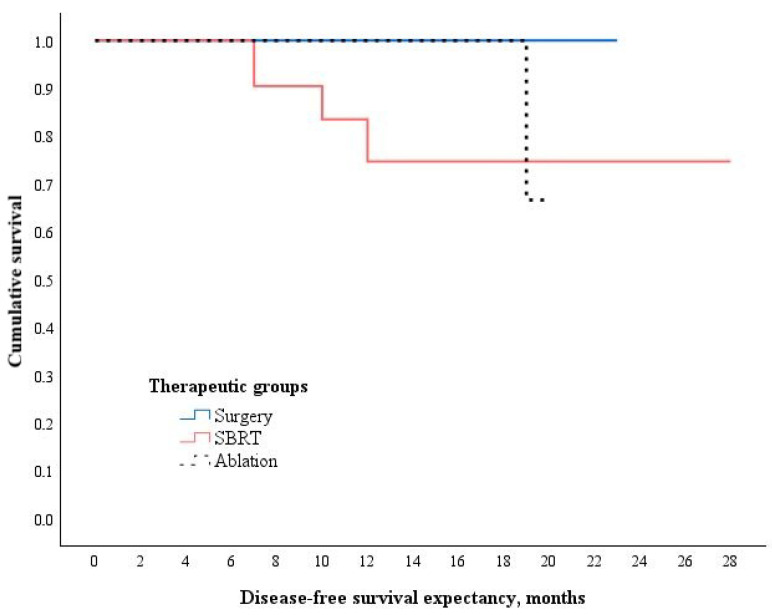
Kaplan–Meier curve for disease-free survival across the therapeutic groups. χ^2^ (2, N = 68) = 6.262, *p* = 0.044; Wilcoxon (Gehan) Statistic.

The mean disease-free survival for the study population was 10.9 ± 5.7 months. Patients who underwent surgery had a significantly longer DFS compared to those treated with SBRT, at 12.4 ± 4.2 months versus 9.9 ± 6.7 months, respectively (*p* = 0.047) (Figure 3). The mean DFS in the ablation group was 10.9 ± 5.5 months, which did not differ significantly from either the surgery group (*p* = 0.279) or the SBRT group (*p* = 0.098).

### 3.4. Complications

The overall complication rate was 42.6%, with statistically significant differences between all treatment groups (*p* < 0.05). The highest rate was observed in the surgery group (78.9%), followed by ablation (45.0%), while SBRT had the lowest complication rate (17.2%). Serious complications were uncommon (n = 8) and occurred slightly more often in the surgery group, though the difference was not statistically significant. Table 6 summarises the distribution of complications across treatment groups.

In the ablation group, eight patients developed pneumothorax: one case was aspirated during the procedure, another one required chest tube insertion for 24 h, and the remaining six resolved spontaneously. In contrast, all surgical patients were managed with two postoperative chest drains. Additionally, one patient in the ablation group developed pneumonia, which was successfully treated with oral antibiotics, making it the only grade 3 complication in this group.

In the SBRT group, one patient was hospitalized for acute respiratory failure, which was classified as a grade 4 complication. Later on, this patient experienced an acute coronary event resulting in death. Two patients were diagnosed with pneumonia or abscess and were treated conservatively with oral antibiotics (both grade 3 complications), while another two experienced worsening shortness of breath following radiation therapy and were managed conservatively without specific intervention.

The surgical group had two grade 4 complications, requiring revision thoracotomy due to necrotizing pneumonia or postoperative bleeding. Additionally, two patients required extended hospitalization due to infection, with one developing pleural empyema, necessitating additional drainage, both classified as grade 3 complications. The remaining 11 patients experienced severe postoperative pain, shortness of breath, and weakness, which were managed conservatively.

### 3.5. Hospitalization

**Figure 4:**
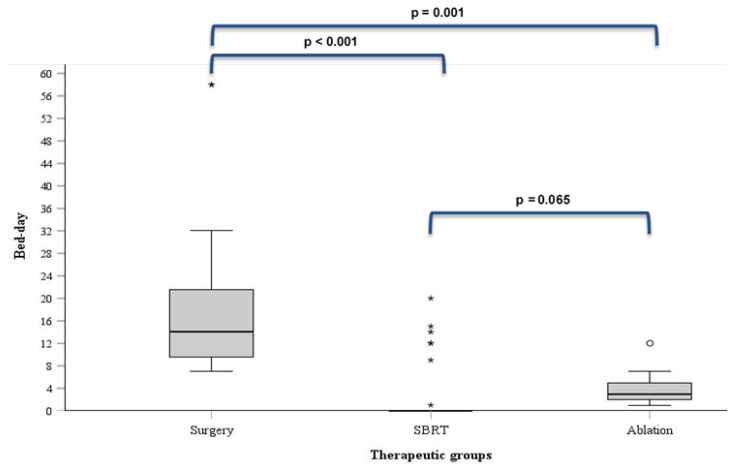
The duration of hospitalization across the therapeutic groups (asterisks (*) and small circles (°) indicate outlier data points).

The median hospital stay was 14 days (IQR: 9–22 days) for the surgery group, 0 days (IQR: 0–0.5 days) for SBRT group and 3 days (IQR: 2–5 days) for ablation group (Figure 4). Surgical patients required significantly longer hospital stays compared to SBRT and ablation groups (both *p* < 0.05), while no such difference was observed in SBRT vs. ablation pair (*p* = 0.065).

Notably, 75.9% of SBRT patients were treated on an outpatient basis, while seven patients required hospitalization due to a poor ECOG performance status (n = 4) or social factors limiting adherence to the treatment plan (n = 3).

### 3.6. Health-Related Quality of Life

Table 7 displays the initial SF-36 questionnaire scores across the treatment groups and their comparisons. Surgery patients had the best overall HRQoL indicator scores, with a marked advantage over SBRT patients in most domains (all except mental health and bodily pain). Ablation patients were generally comparable to surgery patients, except for lower social functioning and role limitations due to emotional health scores. There were no significant differences between SBRT and ablation across all domains.

Treatment-induced changes (Δ, or “delta” values) in physical and psychological health domains within and across all therapeutic groups are displayed in Table 8 and Table 9. Notably, the mean delta values for all domains in the surgery group were negative with a markedly unfavorable impact on physical functioning, role physical and bodily pain. In contrast, patients in the ablation group showed improvement across all domains with significant positive effects on multiple indicators. Six out of eight indicators in the SBRT group also improved post-treatment, particularly mental health and social functioning.

When comparing groups, all HRQoL indicators, except role limitations due to emotional health, were significantly improved in the ablation group compared to surgery. Additionally, in the surgery vs. SBRT comparison, physical functioning, role limitations due to physical health, and social functioning showed significantly better outcomes with SBRT. Yet, SBRT and ablation showed similar post-treatment HRQoL effects.

**Figure 5:**
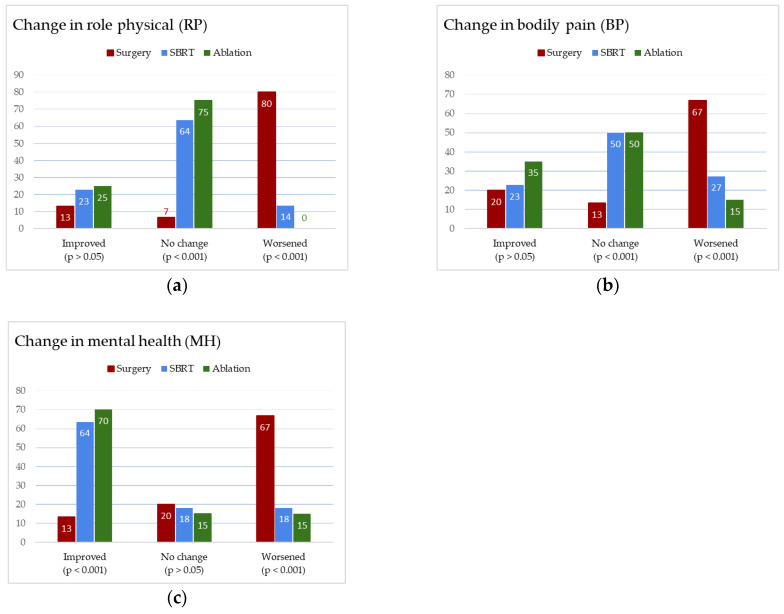
Bar charts: the impact of treatment method on quality-of-life outcomes—role physical (**a**), bodily pain (**b**), and mental health (**c**).

The provided bar charts (Figure 5) compare the effects of three treatment methods—surgery (red), SBRT (blue), and thermal ablation (green)—on three quality-of-life domains: Role Physical (RP), Bodily Pain (BP), and Mental Health (MH). Surgery had the most negative impact across these three HRQoL domains, with the highest percentage of patients experiencing worsening conditions and the lowest improvement rates. SBRT showed mixed outcomes, with the majority of patients reporting no change in bodily pain and role limitations due to physical health. Ablation demonstrated the most favorable results across these indicators, with low rates of patients experiencing worsening and the highest improvement percentages. Notably, changes in mental health were significantly improved in patients treated with SBRT and ablation compared to those who underwent surgery.

Key outcomes showing significant changes among treatment groups are summarized in Table 10.

## 4. Discussion

The three principal curative treatment options for malignant lung lesions are surgery, radiotherapy, and image-guided ablation. While each modality presents distinct advantages and limitations, the selection of therapy is influenced by multiple patient- and tumour-related factors. In certain clinical scenarios, the optimal choice remains a topic of debate. Most institutions continue to recommend surgery as the first-line treatment, reserving radiotherapy for patients who are medically inoperable or decline surgery. Meanwhile, ablation remains less widely adopted, largely due to limited access and clinician experience.

This study evaluated treatment outcomes for malignant lung lesions, encompassing both primary and metastatic cases, which were unevenly distributed across treatment groups. The demographic and clinical profile of the cohort was consistent with typical NSCLC and metastatic lung cancer populations. Surgery and SBRT were primarily used for patients with primary NSCLC, whereas ablation was predominantly applied in those with oligometastatic disease. Given that metastatic lesions were more frequently treated with ablation or SBRT, some of the observed relapses and deaths likely reflect the underlying systemic disease burden. This limits the direct comparisons in survival and recurrence rates across the treatment modalities. SBRT patients were generally older, had a greater cumulative smoking history, and lower ECOG performance scores. However, these differences did not reach statistical significance when compared with the surgery and ablation groups. Nonetheless, such baseline imbalances may represent potential confounding factors, and their influence on overall survival outcomes in this cohort cannot be excluded. Given minimal loss to follow-up, attrition bias was unlikely.

Although the findings of this study reaffirm the efficacy of surgery in achieving disease control, they also underscore the potential drawbacks of major surgical interventions. Within the study cohort, surgical patients self-reported the highest baseline scores across all HRQoL domains. But they experienced the most adverse treatment-related decline in HRQoL, with several domains worsening significantly. Among the treatment groups, surgical patients experienced the highest complication rates, the most severe adverse events, and the longest hospital stays. Notably, SBRT and ablation patients had lower baseline HRQoL scores, which remained stable or improved following treatment. Furthermore, this study demonstrated that image-guided ablation showed comparable outcomes to stereotactic body radiotherapy in terms of disease control, disease-free survival, duration of hospital stay, incidence of significant complications, and preservation of quality of life.

A comprehensive meta-analysis evaluating survival outcomes of surgery versus ablation in stage I NSCLC revealed that surgery provided significantly better short-term (1- and 2-year) DFS, but no significant differences were observed beyond 3 years [21]. Moreover, no significant differences in the 1- to 5-year overall or cancer-specific survival were found between the treatment groups. In support with our results, a few comparative studies reported no significant differences in distant recurrence between lobectomy and MWA among standard-risk and high-risk early-stage NSCLC patients [22,23,24].

Another extensive meta-analysis focusing on early-stage NSCLC demonstrated superior OS in the surgery group compared to SBRT, though local control rates at 3 years were similar [25]. In comparison, Wang Y. and colleagues [26] found that for colorectal cancer pulmonary oligometastases, surgery resulted in significantly better freedom from intrathoracic progression and progression-free survival (PFS) than SBRT, while OS did not differ substantially between the modalities.

In alignment with our observations, a meta-analysis by Chen et al. [27] reported comparable OS, PFS, and rates of severe adverse events between SBRT and local ablation techniques for early-stage NSCLC. However, it is important to note that most of the studies in this meta-analysis assessed radiofrequency ablation. A meta-analysis by Laeseke et al. [28] and a study by Nour-Eldin et al. [29] both demonstrated the superiority of MWA over RFA in terms of disease control and overall clinical outcomes, with MWA showing comparable recurrence rates to SBRT in Laeseke P. et al. study.

For CRC pulmonary metastases, Dong et al. [30] reported that MWA provided superior local disease control and overall survival compared to SBRT. Similarly, Cooke et al. [31] demonstrated strong local tumor control rates following MWA, local progression-free survival of 91.9%, 85.9%, and 81.5% at 1, 2, and 3 years, respectively. The authors concluded that MWA may help prolong chemotherapy-free survival in patients with CRC lung metastases. A recent systematic review and meta-analysis by Laeseke et al. [32], which pooled RFA and MWA outcomes under a single “ablation” category, found that SBRT was associated with higher local tumor progression rates, while overall survival differences were significant at 1 year but not at 2–3 years. Collectively, these findings suggest that MWA, in particular, may offer superior local control and survival benefits over SBRT for malignant lung lesions, and caution is warranted when interpreting conclusions derived from pooled ablation data that include both RFA and MWA.

Managing marginal recurrence remains a significant challenge. Repeat surgery is often technically demanding and may further impair lung function, especially in patients who have previously undergone major resections such as lobectomy (as majority in this study). Many patients treated with SBRT are not surgical candidates, and reirradiation is feasible only in a subset of cases, requiring strict planning to manage toxicity. Published data suggest that repeat SBRT can achieve satisfactory local control and survival outcomes, though occasional severe toxicities reinforce the need for careful patient selection [13]. IGTA, however, avoids these limitations and is generally an excellent treatment option for marginal recurrence [33]. Notably, in our cohort, the majority of local recurrences in the ablation group (5 out of 6) were successfully managed with repeat ablation. This highlights its feasibility and effectiveness as a retreatment strategy. In contrast, reirradiation was performed in one case only (out of 10 local recurrences).

The identification, interpretation and grading of post-procedural complications vary considerably across the literature. In this study, the observed rates of major complications align with the general ranges reported in previous publications [34,35,36]. Major complications were slightly more frequent and severe in the surgery group (21.1%) compared to ablation (10.3%) and SBRT (5.0%). This highlights more intensive postoperative recovery for surgery patients that occasionally require additional invasive interventions, whereas grade ≥ 3 complications following ablation and SBRT were generally managed conservatively. Consistent with our findings, Yao W. et al. [22] reported significantly fewer complications after MWA compared to lobectomy in stage I NSCLC, while Wang Y. et al. [24] found all complications—except pneumothorax—to be more frequent after lobectomy, with significantly higher rates of infection and respiratory failure. Moreover, several studies also report shorted post-treatment hospitalization for ablation versus surgical intervention [21,24].

An important yet often overlooked aspect in determining the optimal treatment strategy is cost, although this was not assessed in our study. While minimally or non-invasive treatments are generally assumed to be more economical, true cost-effectiveness depends on various factors assessed through detailed cost-utility analyses. For instance, Igarashi A. et al. [37] reported that SBRT was a more financially efficient option than surgery for operable stage I NSCLC patients in Japan. Conversely, another study found lobectomy to be more cost-effective than SBRT for standard-risk patients, whereas SBRT was favored over wedge resection in marginally operable cases [38]. Interestingly, Wu X. et al. [39] demonstrated that MWA was more cost-effective than SBRT in inoperable stage I NSCLC patients. Thus, available evidence suggests that SBRT and MWA may offer more cost-effective alternatives to surgery in selected stage I NSCLC populations, depending on operability status and treatment context.

In addition to cancer-related outcomes, quality of life plays a crucial role for patients with malignant lung lesions and can significantly influence treatment decisions. However, HRQoL is often underreported or overlooked in clinical studies, despite its importance in guiding patient-centered care and assessing the true impact of different treatment modalities. Quality of life data for ablation in lung malignancies is scarce. Palussière et al. [40] assessed HRQoL after RFA for stage IA NSCLC using EORTC QLQ-C30 questionnaires, noting increased fatigue and cognitive decline but no significant impact on overall health or quality of life. In contrast, our study found ablation had the most favorable impact across multiple HRQoL indicators.

More robust data exist for surgery and SBRT. Consistent with our findings, Schwartz et al. [41] and Samson et al. [42] observed worse baseline physical and mental quality of life in SBRT patients, likely due to poorer functional status and more comorbidities (as reflected in the disproportion of ECOG scores in our study). However, treatment effects varied: Schwartz et al. [41] reported declines in both groups, whereas Samson et al. [42] found short-term deterioration in role functioning for surgery and improvements in some HRQoL domains after SBRT, which returned to baseline within 6 months—except for emotional functioning, which improved in both groups. These results align with a systematic review concluding that SBRT patients generally experience a stable HRQoL, while surgery often causes a temporary decline with recovery within a year [43]. However, our study did not assess potential later improvements in HRQoL domains.

This study has several limitations in addition to those previously mentioned. It was a single-center, prospective, non-randomized trial with a relatively small patient cohort. Patients recruited to the SBRT or ablation groups were generally those more frail or unfit for surgery—as indicated by their ECOG scores and poorer baseline HRQoL scores—or unwilling to undergo it. While the study aimed to assess treatment outcomes for malignant lung lesions, it did not restrict analysis solely to patients with primary lung cancer, who were unevenly distributed between the treatment groups. Patients with systemic metastatic disease or confirmed recurrence during follow-up were often subjected to systemic therapy. Some of them underwent additional local treatment (primarily repeat ablation or radiotherapy) to maintain disease control and prolong survival. As a result, direct comparisons of treatment effectiveness across the groups are constrained. Outcome stratification by NSCLC lesion T stage was not conducted, as the limited number of T2 lesions precluded a meaningful statistical comparison. In addition, long-term oncologic outcomes were not the primary focus of this study, and given the relatively short median follow-up period, no strong conclusions regarding long-term survival can be drawn. Existing literature presents significant variability in study protocols and imbalanced treatment arm sizes, further complicating reliable comparisons. Future research should involve large, multicenter randomized trials with standardized protocols and stratified patient groups. This will enable more accurate and reliable comparisons of treatment outcomes and quality-of-life across surgical, ablative, and radiotherapeutic modalities for malignant lung lesions.

This study demonstrates that minimally invasive, image-guided ablation can achieve disease control comparable to SBRT while better preserving quality of life. By addressing both oncologic and functional outcomes, our findings underscore the value of interventional radiology in expanding treatment options for patients who are not surgical candidates. These results support the integration of ablation into contemporary cancer care pathways and highlight its potential to strengthen the role of interventional oncology in multidisciplinary management.

## 5. Conclusions

This study demonstrated that minimally invasive image-guided thermal ablation provides comparable disease control and health-related quality of life outcomes to stereo-tactic body radiotherapy, with the added benefit of lower complication rates. While surgi-cal resection achieved the lowest recurrence rates and the longest disease-free survival, it was also associated with the highest incidence of complications, prolonged recovery, and the greatest negative impact on the quality-of-life. In contrast, thermal ablation emerged as a well-tolerated alternative, particularly beneficial for patients with comorbidities or reduced functional status. These findings highlight the potential of thermal ablation as an effective and patient-centered therapy in the multidisciplinary management of malignant pulmonary lesions.

## Figures and Tables

**Table 1:** Dose and fractionation regimens for lesions treated with SBRT.

Dose	Fractions	Number of Lesions
34 Gy	1	1
40 Gy	1	1
45 Gy	3	1
45 Gy	5	2
50 Gy	5	15
54 Gy	3	4
55 Gy	5	4
55 Gy	11	1
60 Gy	8	4

**Table 2:** Baseline dosimetric parameters of lesions treated with SBRT.

Variables	Mean	Median	Range	Standard Deviation
ITV (cc)	13.29	12.5	0.9–47.9	11.06
PTV (cc)	34.32	34	6.2–86.5	21.93
MLD (Gy)	3.06	3.05	1.18–5.95	1.27
V20 (%)	3.99	4.12	0.73–9.26	2.14
V12.5 (%)	6.98	7.79	1.74–15.96	3.43
V5 (%)	13.72	13.02	5.72–27.07	5.53

IVT—internal target volume; PTV—planning target volume; MLD—mean total lung dose; V20—percentage of lung volume (minus ITV) receiving a radiation dose of ≥20 Gy; V12.5—percentage receiving ≥12.5 Gy; V5—percentage receiving ≥5 Gy.

**Table 3:** Baseline characteristics of the patients and their treated lesions.

	Surgery	SBRT	Ablation	Total	*p*-Value
Age, mean ± SD, years	67.8 ± 7.9	72.5 ± 9.5	69.2 ± 8.5	70.2 ± 8.9	0.172
Smoking history, mean ± SD, years	33.5 ± 15.7	41.4 ± 10.2	31.3 ± 16.9	36.3 ± 14.3	0.165
Pack-years, mean ± SD, years	28.5 ± 17.9	40.1 ± 23.9	28.6 ± 16.7	33.4 ± 20.7	0.242
ECOG, number (%)					
0	8 (42.1%)	6 (20.7%)	8 (40.0%)	22	0.092
1	11 (57.9%)	19 (65.5%)	12 (60.0%)	42	
2	0	4 (13.8%)	0	4	
Lesion histology, number	19	33	25	77	
Primary, (%)	18 (94.7%) ^a^	25 (75.8%) ^a^	12 (48.0%) ^b^	55	0.002
Metastatic, (%)	1 (5.3%) ^a^	8 (24.2%) ^a^	13 (52.0) ^b^	22	
Primary					
Adenocarcinoma	16	13	10	39	
Squamous cell carcinoma	2	12	2	16	
Metastatic					
CRC	1	4	3	8	
Melanoma		1	1	2	
Ductal carcinoma		1		1	
Oesophageal carcinoma		1		1	
Oropharyngeal carcinoma		1	1	2	
Hemangioendothelioma			4	4	
Hemangiopericitoma			2	2	
RCC			1	1	
Neuroendocrine carcinoma			1	1	
Lesion size, mean ± SD, mm	19.7 ± 5.8	19.7 ± 6.8	15.8 ± 6.9	18.4 ± 6.8	0.057
Lesion location (%)					
RUL	9 (47.4%)	7 (21.2%)	10 (40.0%)	26	0.367
RML	0	3 (9.1%)	2 (8.0%)	5	
RLL	4 (21.1%)	8 (24.2%)	6 (24.0%)	18	
LUL	2 (10.5%)	11 (33.3%)	5 (20.0%)	18	
LLL	4 (21.1%)	4 (12.1%)	2 (8.0%)	10	

CRC—colorectal cancer; RCC—renal cell carcinoma; RUL—right upper lobe; RML—right middle lobe; RLL—right lower lobe; LUL—left upper lobe; LLL—left lower lobe; different superscript letters (e.g., a, b) indicate significant differences between groups; identical letters (e.g., a, b) indicate no difference.

**Table 4:** The duration of follow-up in the study population.

Follow-Up	Total	Surgery	SBRT	Ablation	χ^2^, df = 2, *p*-Value
Follow-up (days); (median, IQR *)	391 (254–534)	370(316–467)	361 (217.25–520.25)	489.5(375.25–581.5)	χ^2^ = 2.57 *p* = 0.277
Follow-up (months), (median, IQR *)	12 (8–17)	12 (10–15)	11 (7–16.75)	16 (12–19)	χ^2^ = 2.615 *p* = 0.270

* IQR—interquartile range.

**Table 5:** The occurrence and pattern of recurrence among the study participants.

Recurrence	Total	Surgery	SBRT	Ablation	χ^2^, df = 2, *p*-Value
Total recurrence *	43.3% (29/67)	21.1% (4/19) ^a^	57.1% (16/28) ^b^	45.0% (9/20) ^a b^	χ^2^ = 6.04 ^a^, *p* = 0.049
Local recurrence **	28.1% (16/57)	n/a	31.3% (10/32)	24.0% (6/25)	χ^2^ = 0.365 *p* = 0.546
Regional recurrence *	26.9% (18/67)	10.5% (2/19) ^a^	42.9% (12/28) ^b^	20.0% (4/20) ^a b^	χ^2^ = 6.706 ^b^, *p* = 0.039
Distant recurrence *	20.9% (14/67)	10.5% (2/19)	28.6% (8/28)	20.0% (4/20)	χ^2^ = 2.244, *p* = 0.326

* Calculated with respect to the patient; ** calculated with respect to the lesion; n/a—non applicable; different superscript letters (e.g., a, b) indicate significant differences between groups; identical letters (e.g., a, b) indicate no difference.

**Table 6:** Distribution of all and serious (grade ≥ 3) complications between the therapeutic groups.

	Total	Surgery	SBRT	Ablation	χ^2^, df = 2, *p*-Value
Complications, all grades	42.6% (29/68)	78.9% (15/19) ^a^	17.2% (5/29) ^b^	45.0% (9/20) ^c^	χ^2^ = 17.934 ^a^ *p* < 0.05
Complications, grade ≥ 3	11.8% (8/68)	21.1% (4/19)	10.3% (3/29)	5.0% (1/20)	χ^2^ = 2.517 *p* = 0.284

Different superscript letters (e.g., a–c) indicate significant differences between groups.

**Table 7:** Comparison of initial SF-36 questionnaire domains across the treatment groups.

SF-36 Scores	Surgery	SBRT	Ablation	p1	p2	p3
Physical functioning, mean (min; max)	79.0 (45; 100)	52.2 (10; 100)	72.8 (15; 100)	0.014	1.000	0.077
Role physical, mean (min; max)	85.0 (0; 100)	45.7 (0; 100)	57.5 (0; 100)	0.015	0.154	1.000
Role emotional, mean (min; max)	91.1 (33.3; 100)	50.7 (0; 100)	51.7 (0; 100)	0.004	0.006	1.000
Vitality, mean (min; max)	64.3 (45; 85)	45.0 (10; 80)	52.5 (25; 85)	0.029	0.257	1.000
Mental health, mean (min; max)	68.3 (52; 88)	53.2 (20; 100)	58.0 (32; 96)	0.055	0.279	1.000
Social functioning, mean (min; max)	89.0 (45; 100)	51.1 (0; 100)	67.9 (22.5; 100)	<0.001	0.035	0.333
Bodily pain, mean (min; max)	85.0 (32.5; 100)	65.2 (12.5; 100)	78.5 (32.5; 100)	0.064	1.000	0.307
General health, mean (min; max)	46.3 (30; 65)	26.5 (0; 70)	32.8 (15; 75)	0.003	0.067	0.912

p1 = surgery vs. SBRT; p2 = surgery vs. ablation; p3 = SBRT vs. surgery.

**Table 8:** Analysis of the impact of treatment on HRQoL indicator scores within the therapeutic groups (*p*-values).

	Δ PF	Δ RP	Δ RE	Δ VT	Δ MH	Δ SF	Δ BP	Δ GH
Surgery, *p*	**0.002 ⇩**	**0.013 ⇩**	0.340	0.206	0.167	0.056	**0.038 ⇩**	0.179
SBRT, *p*	0.874	0.831	0.325	0.477	**0.026 ⇧**	**0.040 ⇧**	0.472	0.271
Ablation, *p*	0.435	**0.039 ⇧**	0.130	**0.003 ⇧**	**0.016 ⇧**	**0.028 ⇧**	0.092	**0.041 ⇧**

Δ = “delta” or change in domain; PF = physical functioning; RP = role physical; RE = role emotional; VT = vitality; MH = mental health; SF = social functioning; BP = bodily pain; GH = general health. Green text with upward arrows (**⇧**) indicates improvement; red text with downward arrow (**⇩**) indicates worsening.

**Table 9:** Comparison of post-treatment changes in health-related quality of life domains from the SF-36 questionnaire across the study groups.

SF-36 Scores	Surgery	SBRT	Ablation	p1	p2	p3
Δ Physical functioning, mean (min; max)	−15.3 (−30; 10)	2.0 (−15; 55)	1.3 (−35; 25)	0.002	0.001	1.000
Δ Role physical, mean (min; max)	−31.7 (−100; 50)	0 (−100; 50)	11.3 (0; 100)	0.004	<0.001	1.000
Δ Role emotional, mean (min; max)	−11.1 (−100; 33.3)	7.6 (−100; 66.7)	15.0 (−100; 100)	0.357	0.079	1.000
Δ Vitality, mean (min; max)	−6.3 (−30; 25)	3.4 (−25; 45)	13.8 (−15; 45)	0.358	0.002	0.111
Δ Mental health, mean (min; max)	−3.7 (−36; 40)	7.0 (−16; 36)	11.6 (−40; 36)	0.058	0.004	0.950
Δ Social functioning, mean (min; max)	−17.0 (−52.5; 45)	7.0 (−35; 45)	10.9 (−30; 42.5)	0.008	0.006	1.000
Δ Bodily pain, mean (min; max)	−25.5 (−77.5; 67.5)	−4.2 (−67.5; 25)	9.0 (−22.5; 67.5)	0.128	0.005	0.628
Δ General health, mean (min; max)	−4.0 (−25; 35)	3.5 (−30; 40)	8.0 (−25; 35)	0.148	0.021	1.000

Δ = “delta” or change in a domain; p1 = surgery vs. SBRT; p2 = surgery vs. ablation; p3 = SBRT vs. surgery.

**Table 10:** Summary of key outcomes.

Outcome Category	Specific Metric	Surgery	SBRT	Ablation	Significance (*p*-Value/Notes)
Baseline Characteristics	Number of patients	19	29	20	
	Number of lesions	19	33	25	
	Primary vs. metastatic	18/1	25/8	12/13	*p* = 0.002
Recurrence	Total (%)	21.1	57.1	45.0	*p* = 0.049
	Regional (%)	10.5	42.9	20.0	*p* = 0.039
Disease-free survival	Mean DFS (months)	12.4 ± 4.2	9.9 ± 6.7	10.9 ± 5.5	Surgery vs. SBRT *p* = 0.047
Complications	Any-grade (%)	78.9	17.2	45.0	Among all pairs *p* < 0.05
Hospitalization	Median stay (days)	14	0	3	Surgery vs. SBRT & ablation *p* < 0.05
HRQoL (Change, Δ)	Δ PF	−15.3	+2.0	+1.3	Surgery vs. SBRT & ablation *p* < 0.05
	Δ RP	−31.7	0	+11.3	
	Δ SF	−17.0	+7.0	+10.9	
	Δ MH	−3.7	+7.0	+11.6	Surgery vs. ablation *p* = 0.004
	Overall trend	Decline	Mild improvement	Best improvement	

Δ = “delta” or change in domain; PF = physical functioning; RP = role physical; SF = social functioning; MH = mental health.

## Data Availability

The raw data supporting the conclusions of this article will be made available by the authors on request.

## Abbreviations

The following abbreviations are used in this manuscript:

NSCLC	Non-Small Cell Lung Cancer
SBRT	Stereotactic Body Radiation Therapy
IGTA	Image-Guided Thermal Ablation
RFA	Radiofrequency Ablation
MWA	Microwave Ablation
ECOG	Eastern Cooperative Oncology Group
CIRSE	Cardiovascular and Interventional Radiological Society of Europe
ECIO	European Conference on Interventional Oncology
ESOI	European Society of Oncologic Imaging
CRC	Colorectal Cancer
RCC	Renal Cell Carcinoma
DFS	Disease-Free Survival
OS	Overall Survival
PFS	Progression-Free Survival
HRQoL	Health-Related Quality of Life
PF	Physical Functioning
RP	Role Physical
RE	Role Emotional
VT	Vitality
MH	Mental Health
SF	Social Functioning
BP	Bodily Pain
GH	General Health
RUL	Right Upper Lobe
RLL	Right Lower Lobe
LUL	Left Upper Lobe
LLL	Left Lower Lobe
GTV	Gross Tumour Volume
CTV	Clinical Target Volume
IVT	Internal Target Volume
PTV	Planning Target Volume
OAR	Organ-at-Risk
BED	Biological Effective Dose
HU	Hounsfield Unit
CBCT	Cone-Beam CT
FDG-PET	Fluorodeoxyglucose Positron Emission Tomography
IQR	Interquartile Range
SD	Standard Deviation
OR	Odds Ratio
CI	Confidence Interval
